# Study of Radiation Resistance of WO_3_ Microparticles under Irradiation with Heavy Kr^15+^ and Xe^22+^ Ions

**DOI:** 10.3390/nano12172909

**Published:** 2022-08-24

**Authors:** Dauren B. Kadyrzhanov, Artem L. Kozlovskiy, Maxim V. Zdorovets, Ainagul A. Khametova, Dmitriy I. Shlimas

**Affiliations:** 1 Engineering Profile Laboratory, L.N. Gumilyov Eurasian National University, Nur-Sultan 010008, Kazakhstan; 2 Laboratory of Solid State Physics, The Institute of Nuclear Physics, Almaty 050032, Kazakhstan

**Keywords:** WO_3_ microparticles, heavy ions, radiation damage, anisotropic distortion, radiation resistance

## Abstract

In this work, we consider the effect of irradiation with heavy Kr^15+^ and Xe^22+^ ions on the change in the structural and strength properties of WO_3_ microparticles, which are among the candidates for inert matrix materials. Irradiation with heavy Kr^15+^ and Xe^22+^ ions was chosen to determine the possibility of simulation of radiation damage comparable to the impact of fission fragments. During the studies, it was found that the main changes in the structural properties with an increase in the irradiation fluence are associated with the crystal lattice deformation and its anisotropic distortion, which is most pronounced during irradiation with heavy Kr^15+^ ions. An assessment of the gaseous swelling effect due to the radiation damage accumulation showed that a change in the ion type during irradiation leads to an increase in the swelling value by more than 8–10%. Results of strength changes showed that the most intense decrease in the hardness of the near-surface layer is observed when the fluence reaches more than 10^12^ ion/cm^2^, which is typical for the effect of overlapping radiation damage in the material. The dependences obtained for the change in structural and strength properties can later be used to evaluate the effectiveness of the use of refractory oxide materials for their use in the creation of inert matrices of nuclear fuel.

## 1. Introduction

In the modern world, more and more attention is paid to the use of new types of refractory ceramics as inert matrices of nuclear fuel, as well as structural materials for high-temperature nuclear reactors [1,2,3]. Such a high demand for the use of refractory materials is due not only to the ever-increasing needs of mankind in energy resources, the problem of which can be partially solved by increasing the share of nuclear energy but also to the technological process, which consists in the use of new power plants with high efficiency. In such facilities, including high-temperature nuclear reactors, it is planned to increase the efficiency not only by increasing operating temperatures but also by replacing the main nuclear fuel with uranium-free fuel using inert matrices [4,5]. It is assumed that the use of inert matrices based on refractory oxide or carbide ceramics, which are highly resistant to high temperatures and mechanical stress, will increase the degree of burnup of uranium-free nuclear fuel, which will lead to an increase in efficiency [6,7]. This fuel is a mixture of transuranium elements (in particular, plutonium) placed in inert matrices based on oxide or carbide refractory compounds. At the same time, great prospects for a new type of nuclear fuel are assigned to plutonium, the use of which, unlike uranium, does not lead to the accumulation of actinides, which reduces the risk of accumulation of radioactive waste during operation and allows processing of weapons-grade plutonium, which is available in sufficient quantities. However, an increase in efficiency due to the burnup of nuclear fuel imposes additional requirements on the materials of inert matrices, which consist of increased radiation resistance to damage and maintaining resistance to subsequent mechanical impacts [8,9].

As is known, an increase in the degree of nuclear fuel burnup will be accompanied by an increase in the degree of radiation damage and, consequently, their accumulation in inert matrices [10,11,12]. At the same time, long-term irradiation of inert matrices with fission fragments or a neutron flux can lead to the appearance of metastable or nonequilibrium states in the structure, the destabilization of which can lead to the initialization of embrittlement processes or the formation of microcracks in the damaged layer. Moreover, the radiation damage accumulation can lead to the so-called gas swelling process due to the formation of pores and their subsequent filling with implanted ions. Such swelling can lead to destabilization of the inert matrix and its partial destruction, which will adversely affect the entire system [13,14,15]. 

Despite a sufficient number of scientific studies and studies in the field of radiation resistance of ceramic materials considered as candidate materials for inert matrices of nuclear fuel, there are still many unresolved issues in this area [16,17,18]. This is primarily due to a large number of candidate materials, as well as the lack of a unified approach to determining and interpreting the observed radiation damage, as well as their consequences [19,20]. In most cases, when describing the mechanisms of radiation damage, classical ideas are used about the ballistic nature of the energy transfer of incident ions to the nuclear and electronic subsystems of the target, which subsequently transform into thermal energy in very short periods of time, which leads to structural changes [21,22]. However, in recent years, more and more studies in the interpretation of the mechanisms of radiation damage use an integrated approach, which consists of considering several factors, including deformation mechanisms, as well as the dielectric nature of materials [23,24,25]. This approach makes it possible to interpret the observed effects most fully and establish the radiation damage effect on the properties of inert matrices or oxide ceramics more accurately. 

Based on the foregoing, the purpose of this work is to study the radiation resistance of WO_3_ microparticles to irradiation with heavy Kr^15+^ and Xe^22+^ ions with energies of 150 and 230 MeV, respectively, as well as to determine the radiation damage mechanisms depending on the accumulated radiation dose. The use of heavy ions with these energies makes it possible to simulate radiation damage processes comparable to the impact of uranium fission fragments in a nuclear reactor, as well as to compare the degree of radiation damage caused by the accumulation of defects at a given depth [26,27].

## 2. Experimental Methods

**Figure 1:**
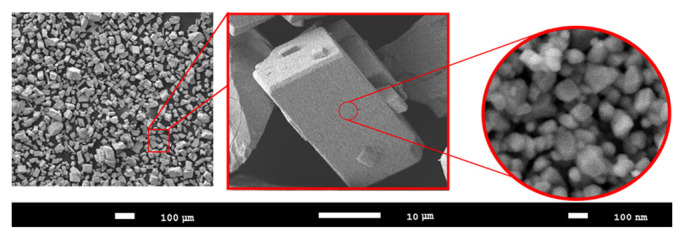
SEM images of the studied WO_3_ microparticles.

The choice of WO_3_ microparticles as objects of study is due to their physicochemical, structural and thermophysical parameters, the combination of which makes them one of the promising materials for the basis of materials for inert nuclear fuel matrices. Samples of WO_3_ microparticles were purchased from Sigma Aldrich (Sigma, MA, USA), and the purity of the samples was ≥99% (PCode 1002551138, CAS 1314-35-8, powder ≤ 25 µm). The dimensions of the samples were determined using scanning electron microscopy. A detailed image of the samples is shown in Figure 1. SEM images were obtained on a Jeol 7500F microscope (Jeol, Tokyo, Japan), and the images were obtained in the SEI mode at an accelerating voltage of 10 kV.

The studied samples are microparticles, the sizes of which vary within 20–25 µm, consisting of spherical grains with a size of 70–100 nm. Such small grain sizes (*D*) indicate a rather high dislocation density, which can lead to a strengthening effect. The relationship between the dislocation density (*l*) and the grain size can be estimated using the expression *l* = 1*/D*^2^, from which it follows that the smaller the grain size, the greater the dislocation density [28,29].

To determine the radiation resistance of WO_3_ microparticles to radiation-induced defects and their accumulation during irradiation. This was caused by interaction with the structure of heavy ions, several experiments were carried out at the DC-60 heavy ion accelerator. Kr^15+^ and Xe^22+^ ions with energies of 150 and 230 MeV, respectively, were chosen as heavy ions, which is due to two possibilities for modeling radiation damage. First, damage and their accumulation depend on the irradiation dose, which is comparable with similar effects in the interaction of ceramics with fission fragments of nuclear fuel. Secondly, with radiation damage caused by neutron irradiation, with one caveat, comparison of damage can only be carried out at a certain depth since neutron radiation has a high penetrating power, unlike heavy ions. According to the calculation of the energy losses of incident ions in WO_3_ microparticles using the SRIM Pro 2013 program code, the values dE/dx_electron_ = 16,000 keV/µm, dE/dx_nuclear_ = 45 keV/µm for Kr^15+^ ions and dE/dx_electron_ = 22,000 keV/µm, dE/dx_nuclear_ = 90 keV/µm for Xe^22+^ ions. The maximum ion path length in microparticles is more than 14 µm for Kr^15+^ ions and more than 18 µm for Xe^22+^ ions. At the same time, the difference between the energy losses of ions during interaction with electron shells and nuclei is more than 2 orders of magnitude, which indicates that the main contribution to changes in structural properties is made by the interactions of incident ions with the electron subsystem of microparticles, and the maximum concentration of implanted ions at the maximum irradiation fluence is not more than 0.001 at.%. According to the estimated calculations, the maximum value of DPA for selected types of ions under irradiation with a fluence of 10^15^ ion/cm^2^ is no more than 0.03–0.035 dpa, which, in terms of neutron flux, is approximately 3–5 × 10^19^ neutron/cm^2^ and is typical for the use of reactor fuel within 1–2 full-fledged campaigns. It is also worth noting that the main changes during irradiation with heavy ions are observed at a depth of the near-surface layer of 14–18 µm, which is subjected to the greatest mechanical stress during fuel operation as well as during deformation processes associated with fuel swelling and destruction.

Irradiation fluences for the structures under study, to simulate the evolution of radiation damage and study their effects on the strength and structural properties, were chosen in the range of 10^11^–10^15^ ion/cm^2^. With these fluences, it is possible to simulate both the effects associated with the formation of single isolated defects and the cases of the formation of cluster defects characteristic of overlapping regions.

The study of structural changes was carried out using the X-ray diffraction method implemented on a D8 Advance ECO X-ray diffractometer (Bruker, Berlin, Germany) powder diffractometer. The X-ray power was 1000 W, Current—25 A, Voltage—40 V, and X-ray Cu-kα wavelength—1.54 Å. The diffraction patterns were taken in the Bragg-Brentano geometry in the range of 2θ = 20–50°, which reflects the main changes in the position and shape of diffraction reflections for WO_3_ structures. For all the studied samples, the time of taking X-ray spectra was the same and amounted to 1 h. All conditions were met in the same mode in order to conduct a comparative analysis of the data obtained and to identify changes in the intensities and shapes of diffraction reflections depending on the irradiation fluence. To determine the phase composition, as well as refine the structural parameters, data from the Crystallography Open Database (REV 173445 2016.01.04) were used.

In the case of the selected types of Kr^15+^ and Xe^22+^ ions, the ion path length is close in magnitude to the transverse dimensions of WO_3_ microparticles, which indicates that the distribution of radiation damage, and, as a result, the deformation distortions caused by them, is equally probable over the entire volume of microparticles. 

The analysis of X-ray data was carried out using the DiffracEVA v.4.2 program code. The refinement of the crystal lattice parameters was carried out using the Nelson-Taylor method [30,31]. In the case of using this technique to calculate the parameters of the crystal lattice and refine them, a system of equations for a monoclinic structure with five unknowns is solved. The solution to this system of equations is embedded in the program code used for calculations and determination of the measurement error. The refinement of the crystal lattice parameters was carried out using the following extrapolation function:

(1) $$f\left(\theta \right)=\frac{1}{2}\left(\frac{cos^{2}\theta }{\theta }+\frac{cos^{2}\theta }{sin\theta }\right),$$

*θ*—diffraction angle.

The use of extrapolation functions makes it possible to use for the refinement of a large amount of experimental data in a wide range of angles *θ*, which, in the case of a monoclinic type of structure, makes it possible to determine the parameters with high accuracy. 

The application of the Williamson–Hall method was used only to evaluate changes in the FWHM value in order to determine the influence of the size and deformation contributions on the change in X-ray peaks. Before taking the studied diffraction patterns, the device was adjusted using a reference Al_2_O_3_ sample, and the comparison of the samples was carried out relative to the studied samples of WO_3_ microparticles in the initial state.

The determination of mechanical properties, in particular, the value of the microhardness of the surface layer and its change as a result of external influences, was carried out using the indentation method implemented using a LECO LM-700 (LECO Corporation, New York, NY, USA) microhardness tester. A Vickers diamond pyramid was used as an indenter; indentation was carried out at an indenter pressure of 50 N.

## 3. Results and Discussion

**Figure 2:**
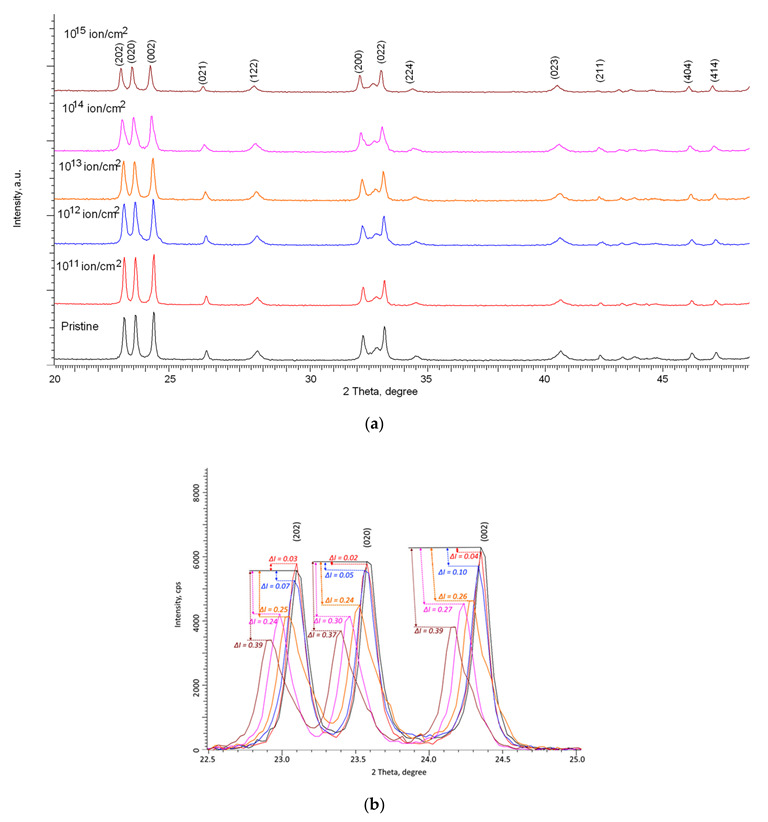
(**a**) Results of X-ray diffraction of the studied samples of WO_3_ microparticles depending on the fluence of irradiation with Kr^15+^ ions; (**b**) Detailed representation of the change in the intensity of the main diffraction reflections depending on the irradiation fluence.

**Figure 3:**
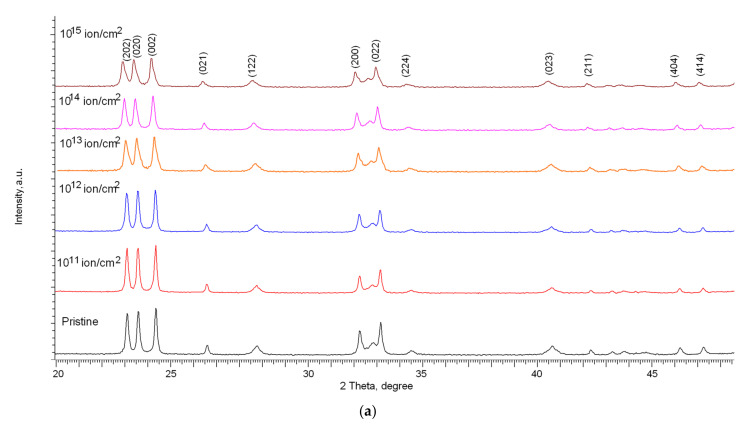

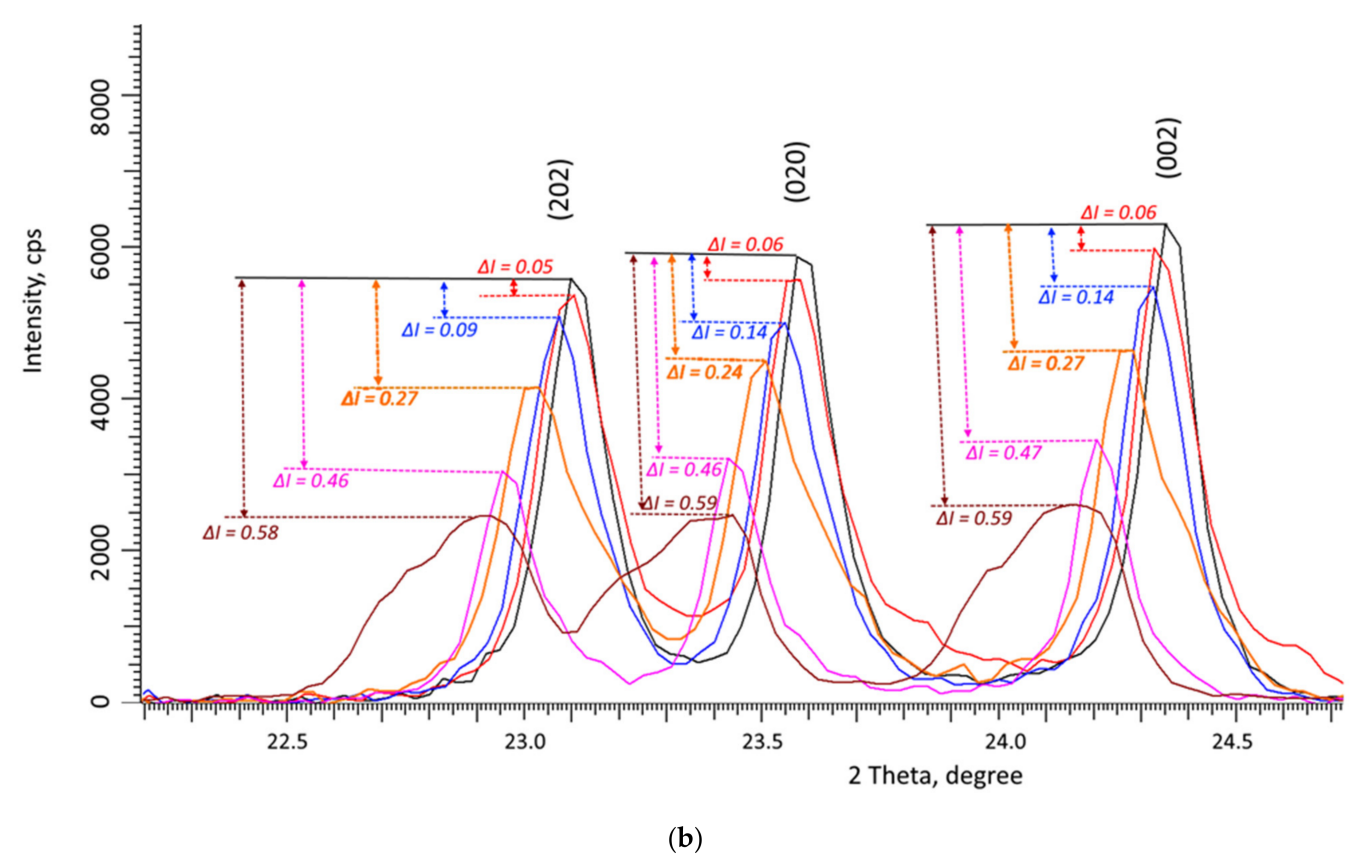
(**a**) Results of X-ray diffraction of the studied samples of WO_3_ microparticles depending on the fluence of irradiation with Xe^22+^ ions; (**b**) Detailed representation of the change in the intensity of the main diffraction reflections depending on the irradiation fluence.

Figure 2 and Figure 3 show the results of measurements of X-ray diffraction patterns of the studied samples depending on the type of incident ions and irradiation fluences. 

The analysis of the obtained diffraction patterns showed that in the initial state the study structures have a monoclinic WO_3_ phase (COD–2311041) (Syngony P121/c(14)) with the crystal lattice parameters of a = 7.6941 Å, b = 7.5434 Å, c = 10.5377 Å, β = 136.61° (these parameters correspond to a distorted crystal lattice along the c axis, which leads to such a large value of the parameter β, which most accurately describes the position of the diffraction maxima of the objects under study). The reference values of the crystal lattice parameters for the monoclinic structure of WO_3_, according to the COD card-2311041, a = 7.6880 Å, b = 7.5390 Å, c = 10.5150 Å, β = 136.06°. It should be noted that the probability of coincidence of the position of the main reflections with the reference values for the COD-2311041 card, taking into account the deformation of the crystal lattice, was more than 90%, while for the structure with the orthorhombic type of the WO_3_ crystal lattice (COD-2107312, a = 7.5700 Å, b = 7.3410 Å, c = 7.7540 Å), the probability of coincidence was less than 80%, in view of which the analysis of structural parameters was carried out taking into account the values characteristic of a monoclinic structure with a spatial syngony P121/c(14). As noted in the work, the WO_3_ structure belongs to the ReO_3_ type, consisting of [WO_6_] octahedra connected at their vertices, and the presence of polymorphism in WO_3_ is due to the mutual rotation of the octahedra or their distortion associated with the displacement of tungsten atoms. At the same time, the main motif of the WO_3_ crystal structure, due to its features and the bound framework of the [WO_6_] octahedra, determines the resistance of the structure to polymorphic transformations or modifications as a result of external influences [32,33].

At the same time, according to the presented X-ray diffraction patterns, no new peaks are observed for the irradiated samples, which indicates the absence of initiation of polymorphic transformation processes as well as the formation of impurity inclusions. The main changes in the diffraction patterns are associated with distortions in the shape of diffraction reflections as well as their displacement, which indicates the deformation of the structure as a result of external influences. Deformation processes can be initiated by the processes of interaction of incident ions as well as the subsequent transformation of kinetic energy into thermal energy in fairly short time intervals. Such a transformation can lead to the appearance of local regions with a strongly changed electron density, which is in a metastable state. At the same time, in the case of low irradiation fluences, these regions are isolated and located at a sufficiently large distance from each other, which can lead to partial relaxation and removal of deformation stresses over time. As a result of this, at irradiation fluences of 10^11^–10^12^ ion/cm^2^, according to the presented X-ray diffraction patterns, an insignificant decrease in the intensity of diffraction reflections is observed, as well as a small shift in the position of diffraction maxima relative to their initial positions.

The calculated data of energy losses, as well as the literature data of several works on radiation damage simulation in oxide and nitride ceramics, indicate that the dimensions of isolated damaged regions that form along the ion trajectory in the material range from 1 to 10 nm, depending on the energy and type of incident ions. In this case, considering the given sizes of the damaged areas, it is possible to make estimates that at irradiation fluences above 10^12^ ion/cm^2^, the beginning of the effect of overlapping defective areas is observed, which leads to the exclusion of the defect isolation effect. 

According to the presented data on X-ray diffraction patterns, at irradiation fluences above 10^12^ ion/cm^2^, the change in the shape and position of diffraction reflections becomes more pronounced than at lower fluences. At the same time, these changes have a pronounced dependence on the type of ions. 

For samples irradiated with Kr^15+^ ions, the main changes in X-ray diffraction patterns are observed at irradiation fluences above 10^13^ ion/cm^2^ and consist of a change in the intensity of diffraction reflections as well as their broadening and shift to the region of small angles, which is characteristic of crystal lattice distortions. At the same time, at fluences of 10^14^–10^15^ ion/cm^2^, a strong asymmetry of the shape of diffraction reflections relative to the position of the maximum is observed, indicating that amorphous inclusions or disordered regions associated with the radiation damage accumulation are formed in the structure. It should also be noted that no obvious changes in the ratio of the intensities of diffraction reflections were observed, which indicates the absence of processes of texture reorientation or strong anisotropy of the crystallite shape under the action of irradiation.

In the case of samples irradiated with Xe^22+^ ions, the changes in the diffraction patterns are already more pronounced at an irradiation fluence of 10^13^ ion/cm^2^, and at the maximum irradiation fluence, a strong distortion and asymmetry of the shape of diffraction reflections and their displacement are observed, which indicates a strong deformation and amorphization of the damaged layer as a result of an increase in the areas of defect overlap as well as an increase in the concentration of the defective fraction.

From the data of a comparative analysis of changes in the intensities of the main diffraction reflections (202), (020), and (002) for both types of irradiations (see the data in Figure 2 and Figure 3), it can be seen that the greatest decrease in the intensity of reflections is observed at irradiation fluences above 10^12^ ion/cm^2^. In this case, the change in the intensity of diffraction reflections (∆*I*) is more than 20% compared to the initial value, and the maximum decrease in intensity in the case of irradiation with Kr^15+^ ions at the maximum fluence is about 40%, while for irradiation with Xe^22+^ ions, this change exceeds 55%. The change in the intensity of the (202), (020), and (002) diffraction reflections in the case of irradiation with Kr^15+^ ions indicates that the deformation of the crystal structure is not isotropic, associated both with the accumulation of deformation distortions and with texture reorientation processes, which is evidenced by the difference in the change in the value of ∆*I* for these reflections depending on the irradiation fluence. In the case of irradiation with heavy Xe^22+^ ions, these changes are less pronounced, and the change in the value of ∆*I* for the three reflections (202), (020), and (002) is approximately the same, which indicates an isotropic distortion of the structure as a result of the accumulation of deformation distortions.

**Figure 4:**
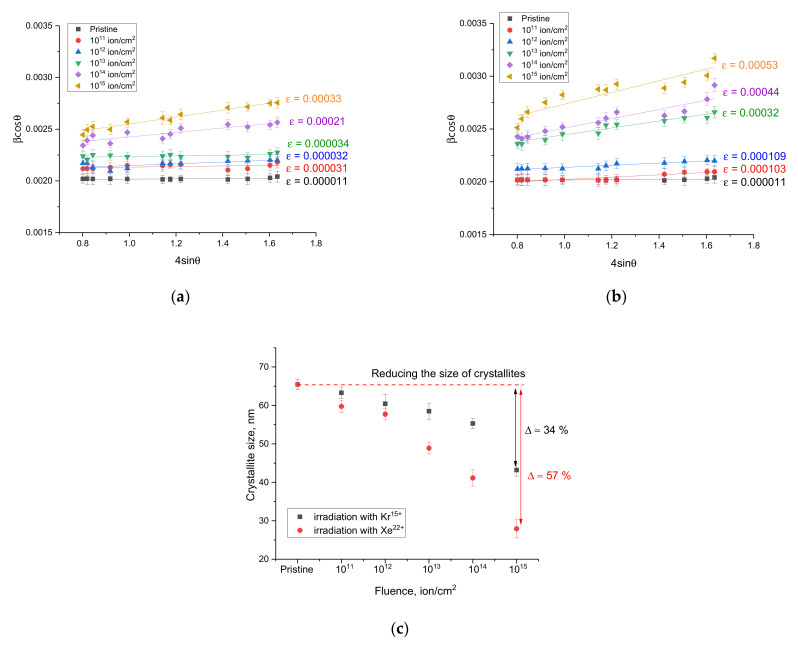
The results of Williamson–Hall plots, reflecting the change in the amount of distortion of diffraction reflections as a result of external influences: (**a**) irradiation with Kr^15+^ ions; (**b**) irradiation with Xe^22+^ ions; (**c**) The results of the change in the size of crystallites as a result of irradiation.

Figure 4 shows the results of the Williamson–Hall plots, characterizing changes in the size of crystallites as a result of external influences, as well as distortions and deformations formed in the structure during interaction with incident ions and the consequences caused by them.

Figure 4c shows the results of the change in the size of crystallites, determined from the constructions of Williamson–Hall, which reflect the processes associated with the fragmentation of crystallites as a result of radiation damage and the consequences caused by them. As can be seen from the presented data, the most pronounced changes in the size of crystallites are observed for irradiation fluences above 10^12^ ion/cm^2^, which consist in a decrease in the size of crystallites, which is associated with recrystallization processes as a result of the accumulation of radiation damage. In this case, the changes are more pronounced for the case of irradiation with Xe^22+^ heavy ions, for which the maximum size reduction is more than 50% at an irradiation fluence of 10^15^ ions/cm^2^.

The general form of changes can be divided into two types associated with a size and deformation effects caused by irradiation. The first type of change is associated with an increase in the FWHM values, which indicates a change in the size of the crystallites and the processes of recrystallization and crushing, leading to their decrease under the action of irradiation. At the same time, in the case of irradiation with Kr^15+^ ions, these effects begin to manifest themselves at irradiation fluences of 10^14^–10^15^ ion/cm^2^, while in samples irradiated with Xe^22+^ ions these changes are observed at a fluence of 10^13^ ion/cm^2^, and the nature of the changes is more pronounced, which indicates that fragmentation of crystallites is more intense. In the case of irradiation with fluences of 10^11^–10^12^ ion/cm^2^, no significant changes are observed, which indicates small changes in crystallites under these irradiation conditions. The second type of change is associated with the broadening of the FWHM value with an increase in the diffraction angle, which indicates the formation of deformation and distortions in the structure. Having estimated the slope of the approximating straight lines that characterize the value of microstrains, it was found that in the case of irradiation with fluences of 10^11^–10^12^ ion/cm^2^, no change in the value of microstrains is observed, from which it follows that the change in FWHM at these irradiation fluences is due only to side effects. At irradiation fluences above 10^13^ ion/cm^2^, both size effects associated with grain fragmentation and deformation effects due to the formation of disordered regions in the structure and an increase in the defect fraction contribute to the change in the FWHM value.

Table 1 presents the results of estimating the crystal lattice parameters of the samples under study depending on the type of external influences. The parameters were estimated using the DiffracEVA 4.2 program code. 

The general trends in the change in the crystal lattice parameters depending on the type of external influence indicate tensile or swelling deformation, and the nature of the change has a strongly pronounced dependence on the irradiation fluence. At the same time, an increase in the irradiation fluence in both cases leads to an increase in the contribution of the crystal lattice deformation, and these changes are more pronounced for irradiation fluences of 10^14^–10^15^ ion/cm^2^. 

**Figure 5:**
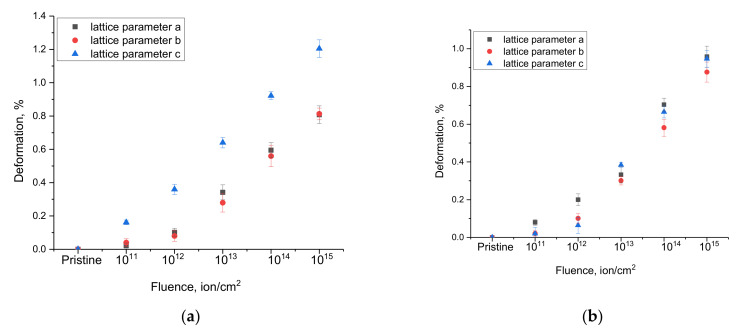
Change in the crystal lattice deformation value as a result of external influences: (**a**) irradiation with Kr^15+^ ions; (**b**) irradiation with Xe^22+^ ions (the amount of deformation is shown in %).

Figure 5 shows the results of the analysis of changes in the crystal lattice deformation along the axes depending on the irradiation fluence. The crystal lattice deformation value was estimated from the change in the ratio of the crystal lattice parameters for each crystallographic axis, which makes it possible to estimate the isotropy of the crystal lattice deformation as a result of external influences. The calculation of the crystal lattice deformation was carried out using the following expression:

$$Deformation=\frac{Lattice\;parameter_{irradiated}-Lattice\;parameter_{initial}\;}{Lattice\;parameter_{initial}}\times 100\%$$

where *Lattice parameter_initial_* and *Lattice parameter_irradiated_* are the crystal lattice parameters in the initial and irradiated states for each axis.

As can be seen from the data presented, in the case of irradiation with heavy Kr^15+^ ions, a pronounced anisotropic distortion of the crystal lattice parameters along the c axis is observed, which indicates the appearance of a distorted deformation of the crystal lattice associated with the radiation damage accumulation. In the case of irradiation with heavy Xe^22+^ ions, the anisotropic distortion of the crystal lattice has a more complex form than in the case of irradiation with heavy Kr^15+^ ions. At low fluences of irradiation with heavy Xe^22+^ ions, anisotropic distortion is observed along the a-axis, while an increase in the irradiation fluence above 10^13^ ion/cm^2^ leads to an increase in anisotropic distortion already along two axes, *a* and *c*. Such a behavior of changes in the crystal lattice deformation in the case of irradiation with heavy Xe^22+^ ions can be due to the effect of a larger number of formed point and vacancy defects due to large energy losses, as well as stronger changes in the electron density, which have a negative effect on the deformation distortions of the crystal structure. 

**Figure 6:**
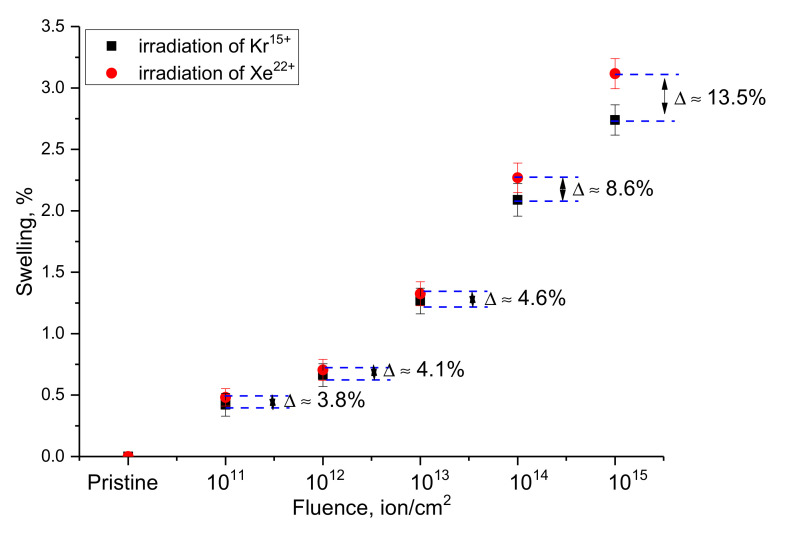
Results of the change in the crystal lattice volume depending on the irradiation fluence (the ∆ value reflects the difference (%) in the swelling value in the case of irradiation with different ions at the same irradiation fluence).

Figure 6 shows the results of a comparative analysis of the crystal lattice swelling of WO_3_ microparticles depending on the type of external influence. This value was estimated from the change in the volume of the crystal lattice of the samples under study before and after exposure to ionizing radiation. The crystal lattice swelling value reflects the tensile strain caused both by the effects of radiation-induced damage accumulation and by ion implantation, which leads to gaseous swelling at high concentrations of implanted ions in the damaged layer. In the case of selected irradiation fluences and energies of incident ions, the main contributions to structural changes and strength properties are made by radiation-induced damage, the formation of vacancy defects and interstices, the formation of which is due to the interactions of incident ions with the crystal structure. According to the estimate of the concentration of implanted ions at these fluences, this value is less than 0.001%, which indicates that the effect of ion implantation in the case of the observed effects is minimal.

The general form of changes in the swelling value depending on the irradiation fluence for both types of ions is close to an exponential dependence, describing these changes depending on the irradiation fluence. In the case of low irradiation fluences of 10^11^–10^12^ ion/cm^2^, the swelling value is no more than 0.5–0.6% of the initial value, which indicates that the formed local defect regions have little effect on the crystal lattice swelling and its deformation. An increase in the irradiation fluence to 10^13^ ion/cm^2^, accompanied by the formation of defect overlap regions, leads to an almost twofold increase in the swelling value. At the same time, it should be noted that an increase in the irradiation fluence leads to an increase in the difference in the swelling values for different types of ions. This behavior can be due to an increase in deformation contributions as well as to the anisotropic nature of the swelling of the crystal lattice, which manifests itself with an increase in the irradiation fluence. 

In the case of irradiation fluences of 10^14^–10^15^ ion/cm^2^, for which, according to the assessment of structural changes, the formation of amorphous inclusions is observed, and the swelling value is more than 2.3–3.5%. At the same time, the difference in the results of swelling for different types of ions also increases, which is due to an increase in the concentration of the defective fraction and a more intense decrease in the crystallinity degree for samples irradiated with Xe^22+^ ions.

A general analysis of the swelling value indicates that at low irradiation fluences, crystal lattice swelling occurs only due to local defect regions or structural distortions caused by them, leading to crystal structure deformation. In the case of high irradiation fluences, swelling is associated both with the effects of anisotropic deformation of the structure and the formation of amorphous inclusions or strongly deformed structural regions in the damaged layer, the presence of which has a pronounced dependence on the type of incident ions and their energy losses. 

According to the data presented in [26], for heavy ions with energies above 100 MeV, the values of nuclear and electronic losses have significant differences, and the value of electronic losses is several orders of magnitude greater than the value of nuclear losses. From this, it follows that the greatest contribution to the change in structural properties is made by changes associated with ionization processes and changes in electron density. In the case of WO_3_ thin films, as shown in [34,35], at ion energies above 100 MeV, irradiation with a fluence of 10^12^ ion/cm^2^ and higher leads to a sharp decrease in the intensity of diffraction reflections as well as a change in their shape, which is close to amorphous. Such a change in diffraction reflections indicates the processes of destruction and partial amorphization associated with a change in the electron density, which, as shown in these studies, is strongly distorted with an increase in the irradiation fluence. At the same time, for thin films, the processes of destruction and amorphization during irradiation are more pronounced than for microparticles, which is due to the structural features of thin films as well as the method of their preparation, which is accompanied by the formation of an initial strongly deformed structure (results of X-ray diffraction of the initial samples). Moreover, an important role in the destruction processes is played by the crystallite sizes, which in the case of thin films are quite small, which leads to a large dislocation density, the change of which leads to the acceleration of amorphization processes and affects the change in mechanical properties, as evidenced by the results of [36]. In the case of microparticles, in which, according to X-ray diffraction data, the value of deformation distortions in the initial state is rather low, irradiation with heavy ions at low fluences does not lead to such serious changes as in the case of thin films. The most pronounced changes are manifested in the case when the effect of overlapping defective areas along the ion motion trajectory in the material occurs. Moreover, the greater the energy of the incident ions, the greater the radius of the local damage area, and as a result, the occurrence of the overlap effect occurs at lower fluences. In the case when local defect regions with a changed electron density and the presence of point defects are isolated, structural changes are due to small distortions associated mainly with the formation of point defects that have little effect on the crystal structure deformation (see results in Figure 5 and Figure 6). In the case of irradiation with high fluences, an effect can be observed associated with the overlap of defective regions that appear along the trajectory of the incident particles in the material, which, at low irradiation fluences, are isolated from each other (the so-called latent tracks, which are well manifested in polymeric materials [37]). When defective areas overlap or merge, highly disordered areas can form in the damaged layer, which contains deformation distortions and stresses, the accumulation of which can lead to the destabilization of strength and hardness [18,19]. An increase in the concentration of such inclusions with an increase in the irradiation fluence can lead to the destruction of the damaged layer and the deterioration of its strength characteristics [21,22]. 

Analyzing the obtained changes in the deformation of the axes of the crystal lattice and its swelling (volume change), we can draw the following conclusions and assumptions about the causes of the observed effects. When analyzing the change in the crystal lattice volume, it was found that an increase in the irradiation fluence leads to an increase in volume, which indicates processes associated with the formation of radiation defects that can cause crystal lattice deformation. At the same time, the magnitude of the change in the crystal lattice volume is an integral value that reflects the overall changes in volume as a result of external influences. However, by analyzing the changes in parameters along each crystallographic axis, it was found that these changes are anisotropic in nature in deformation, which is typical for structures with a non-cubic crystal lattice. In itself, the anisotropic nature of the change in deformation indicates that the distortion of the crystal lattice is not uniform, and at high fluences, it was expressed along the *c* axis during irradiation with Kr^15+^ ions, and in the case of irradiation with Xe^22+^ ions, deformation processes are close to the isotropic nature of deformation distortions. Based on this, it can be concluded that deformation processes have a pronounced dependence on the type and energy of incident ions, while the swelling value reflects the general form of structural distortions of the crystal lattice volume as a result of external influences.

In fact, under irradiation with heavy ions, the main role in the deformation processes and consequences associated with radiation damage is played by the effects associated with the formation of vacancies and interstices, as well as their accumulation and agglomeration with an increase in the fluence or ion energy. At the same time, at low irradiation fluences, most of the resulting radiation defects annihilate, which does not lead to significant structural changes, as evidenced by a number of works [12,13,14,15,16,17,18,19,20]. In the case of irradiation fluences above 10^12^ ion/cm^2^, a stage of radiation damage accumulation is observed due to the effect of overlapping areas of radiation damage occurring along the trajectory of ions in the material, which leads to the formation of cluster defects that can significantly affect the change in the structural and strength properties of the irradiated material. In turn, the magnitude of the crystal lattice deformation and the change in its volume (swelling) directly reflect the dependence of the change in the concentration of radiation-induced defects and their effect on structural distortions.

**Figure 7:**
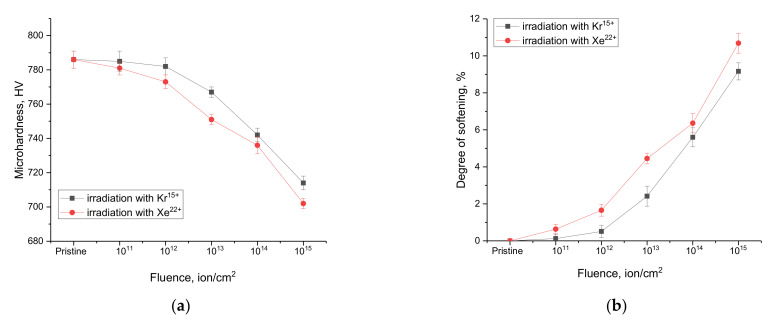
(**a**) Results of the change in the microhardness value depending on the irradiation fluence; (**b**) Results of the change in the softening degree.

Figure 7 shows the results of changes in the microhardness of samples of WO_3_ microparticles depending on the irradiation conditions. These changes characterize the surface layer softening effects associated with radiation damage accumulation as well as deformation effects, leading to a decrease in the resistance of materials to external influences. 

The general view of changes in the microhardness value, which is characteristic of the damage depth at a distance of 0.5–1.5 µm from the surface, indicates the cumulative effect of radiation damage, which, with its increase, has a negative effect on the decrease in the strength characteristics of microparticles. 

As can be seen from the data presented, in the case of irradiation with heavy Kr^15+^ ions, the most pronounced changes in microhardness are observed for fluences above 10^12^ ion/cm^2^, which is due to the effect of overlapping defective regions, accompanied by radiation damage accumulation, as well as the occurrence of anisotropic deformation of the crystal lattice, which is more pronounced in the case of irradiation with Kr^15+^ ions. An increase in the irradiation fluence above 10^12^ ion/cm^2^ leads to a decrease in the resistance of the damaged near-surface layer to mechanical stress by approximately 2–3% with an increase in the fluence by one order of magnitude. Such a change indicates the cumulative effect of damage and the softening of the near-surface layer. 

In the case of irradiation with heavy Xe^22+^ ions, noticeable changes in microhardness are already observed at a fluence of 10^11^ ion/cm^2^, which may be due to the fact that with a change in the ion type and energy, the sizes of local damaged regions that appear along the trajectory of ions in the material are larger, which leads to the apparent effect of overlapping defective regions at lower fluences than in the case of irradiation with Kr^15+^ ions. At the same time, the analysis of structural changes for the case of irradiation with heavy Xe^22+^ ions showed that an increase in the irradiation fluence during irradiation with heavy Xe^22+^ ions lead to greater structural distortions than in the case of irradiation with heavy Kr^15+^ ions. 

The obtained results of changes in the microhardness of the damaged layer indicate the resistance of WO_3_ microparticles to radiation damage depending on the irradiation fluence and, as a consequence, to the accumulation of radiation defects and the structural distortions caused by them. The results indicate that at maximum irradiation fluences, the maximum softening of microparticles is no more than 10%, which is quite acceptable for inert matrix materials during long-term operation. Taking into account the fact that, under real operating conditions, such radiation damage values accumulate for a rather long time, we can conclude that it is promising to use WO_3_ microparticles as materials for inert nuclear fuel matrices.

## 4. Conclusions

The paper presents the results of experimental work related to the study of the radiation resistance of WO_3_ microparticles to irradiation with heavy Kr^15+^ and Xe^22+^ ions. The main research method was the X-ray diffraction method, which allows us to determine the structural changes in materials as well as the factors causing these changes, and the indentation method, which determine the microhardness of the damaged layer. During the studies carried out, the dependences of the change in structural parameters and the effect of crystal lattice swelling were established depending on the irradiation fluence and the type of incident ions. The established anisotropic nature of deformation distortions of the crystal lattice, according to the data obtained, is more pronounced for irradiation with heavy Xe^22+^ ions, while for irradiation with heavy Kr^15+^ ions, anisotropic distortion is observed only along one crystallographic axis. An analysis of the strength properties of the studied WO_3_ microparticles showed that the most intense decrease in the hardness of the near-surface layer is observed when the fluence reaches more than 10^12^ ions, which is typical for the effect of overlapping radiation damage in the material.

Analyzing the obtained data on structural and strength changes, we can draw the following conclusions regarding the mechanisms of radiation damage depending on the type of incident ions. In the case of irradiation with heavy Kr^15+^ ions, an increase in the irradiation fluence leads to an anisotropic distortion of the crystal structure, expressed by an increase in lattice deformation along the c axis, which indicates the accumulation of radiation defects and their effect on structural distortions. In this case, the accumulation of defects occurs due to the formation of the effect of overlapping damaged areas, which leads to the formation of cluster defects due to their agglomeration. 

In the case of irradiation with heavy Xe^22+^ ions, the effects associated with radiation damage are more pronounced, and the effect of anisotropic distortion of the crystal lattice is less pronounced, which is due to the fact that structural changes are more significant during irradiation with heavy Xe^22+^ ions, especially in the case of high irradiation fluences. 

Moreover, in view of the fact that during most of the movement of incident ions in the material of microparticles, electronic losses of ions dominate, the main mechanisms of defect formation are associated with the processes of ionization and subsequent changes in the electron density, which lead to the destruction of crystalline and chemical bonds. The effect of implantation in the case of irradiation fluences of 10^11^–10^15^ ion/cm^2^ on the crystal structure disorder degree and the decrease in strength properties is minimal due to the low probability of implantation of ions and their accumulated concentration.

## Figures and Tables

**Table 1:** Lattice parameter data.

Irradiation Fluence, Ion/cm^2^	Irradiation with Kr^15+^ Ions		Irradiation with Xe^22+^ Ions	
	Crystal Lattice Parameter, Å	Crystal Lattice Volume, Å^3^	Crystal Lattice Parameter, Å	Crystal Lattice Volume, Å^3^
Initial sample	a = 7.6941 ± 0.0024, b = 7.5434 ± 0.0015, c = 10.5377 ± 0.0018, β = 136.61 ± 0.22°	422.55 ± 0.19	a = 7.6941 ± 0.0024, b = 7.5434 ± 0.0015, c = 10.5377 ± 0.0018, β = 136.61 ± 0.26°	422.55 ± 0.19
10^11^	a = 7.6958 ± 0.0016, b = 7.5464 ± 0.0012, c = 10.5756 ± 0.0019, β = 136.63 ± 0.31°	424.33 ± 0.32	a = 7.7003 ± 0.0016, b = 7.5448 ± 0.0019, c = 10.5318 ± 0.0017, β = 136.64 ± 0.27°	424.59 ± 0.25
10^12^	a = 7.7019 ± 0.0022, b = 7.5495 ± 0.0015, c = 10.5883 ± 0.0016, β = 136.67 ± 0.34°	425.35 ± 0.16	a = 7.7095 ± 0.0022, b = 7.5510 ± 0.0021, c = 10.5444 ± 0.0026, β = 136.68 ± 0.25°	425.53 ± 0.27
10^13^	a = 7.7204 ± 0.0016, b = 7.5645 ± 0.0021, c = 10.6052 ± 0.0017, β = 136.68 ± 0.25°	427.90 ± 0.68	a = 7.7197 ± 0.0018, b = 7.5661 ± 0.0022, c = 10.5782 ± 0.0016, β = 136.71 ± 0.32°	428.15 ± 0.53
10^14^	a = 7.7399 ± 0.0015, b = 7.5856 ± 0.0022, c = 10.6349 ± 0.0017, β = 136.69 ± 0.22°	431.38 ± 0.74	a = 7.7483 ± 0.0015, b = 7.5873 ± 0.0021, c = 10.6078 ± 0.0024, β = 136.74 ± 0.31°	432.14 ± 0.86
10^15^	a = 7.7563 ± 0.0015, b = 7.6048 ± 0.0014, c = 10.6646 ± 0.0018, β = 136.72 ± 0.23°	434.17 ± 1.11	a = 7.7678 ± 0.0016, b = 7.6095 ± 0.0015, c = 10.6375 ± 0.0021, β = 136.81 ± 0.24°	435.72 ± 1.16

## Data Availability

Not applicable.
